# Finding disease candidate genes by liquid association

**DOI:** 10.1186/gb-2007-8-10-r205

**Published:** 2007-10-04

**Authors:** Ker-Chau Li, Aarno Palotie, Shinsheng Yuan, Denis Bronnikov, Daniel Chen, Xuelian Wei, Oi-Wa Choi, Janna Saarela, Leena Peltonen

**Affiliations:** 1 Department of Statistics, UCLA, 8125 Math Sciences Bldg, Los Angeles, California 90095-1554, USA; 2 Institute of Statistical Science, Academia Sinica, Academia Road, Nankang, Taipei 115, Taiwan; 3 The Finnish Genome Center and Department of Clinical Chemistry, University of Helsinki, Haartmaninkatu, 00290 Helsinki, Finland; 4 The Broad Institute of Harvard and MIT, Cambridge Center, Cambridge, Massachusetts 02142, USA; 5 Department of Pathology and Laboratory Medicine, Gonda Researach Center, UCLA, Los Angeles, California 90095-1766, USA; 6 Department of Human Genetics, UCLA, 695 Charles E. Young Drive South, Los Angeles, California 90095-1766, USA; 7 National Public Health Institute, Helsinki, Finland, Biomedicum Helsinki, Haartmaninkatu, 00290 Helsinki, Finland; 8 Department of Medical Genetics, University of Helsinki, Biomedicum Helsinki, Haartmaninkatu, 00290 Helsinki, Finland

## Abstract

A novel approach to finding candidate genes by using gene expression data through liquid association is developed and used to identify multiple sclerosis susceptibility candidate genes.

## Abstract 2

A novel approach to finding candidate genes by using gene-expression data has been developed and used to identify a multiple sclerosis susceptibility candidate genes.

## Background

Studies aiming to identify susceptibility genes in complex diseases have proceeded along two lines. The traditional candidate gene approach is limited by our ability to come up with a comprehensive list of biologically related genes. On the other hand, the 'hypothesis free' approach relies on genome-wide scans for disease loci, typically via linkage in exceptionally large families or via association in case control studies. Multiple sclerosis (MS), which is one of the most common neurologic disorders affecting young adults, is characterized by demyelination and reactive gliosis [1]. Analogous to many complex traits, genome scans in MS have identified numerous chromosomal loci often with only a nominal evidence for linkage to MS [2-6]. With the notable exception of the human leukocyte antigen (major histocompatibility complex [MHC]) locus on 6p21, evidence for specific MS genes emerging from these studies is still scanty. Thus far, the only associated non-HLA genes replicated in multiple populations are the *PRKCA *gene [7] and the recently reported *IL2RA *and *IL7R *genes [8]. For MS, as for most complex traits, the loci derived from linkage scans have remained quite wide because of multiple uncertainties concerning the disease model in statistical analyses. To expedite the process of gene identification in these wide DNA regions, we need novel approaches to identify potentially involved pathways and to prioritize genes on identified loci for further sequencing efforts.

**Figure 1:**
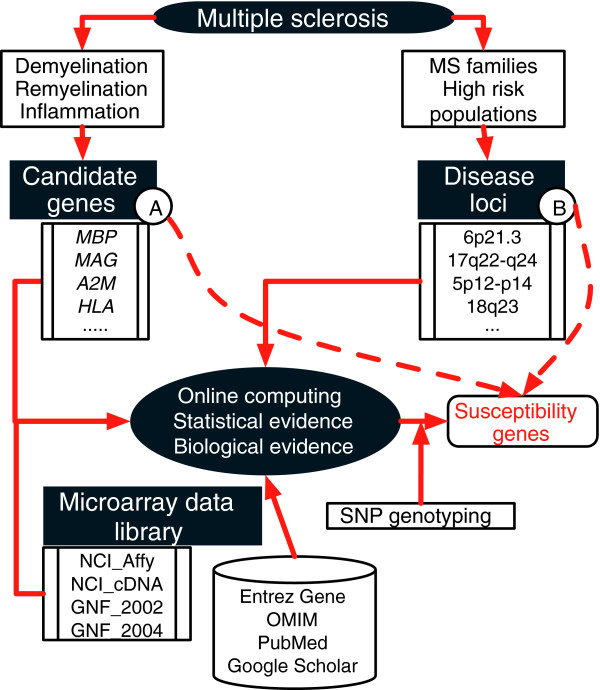
Federated functional genomics approach. The two dashed lines in this diagram indicate **(a) **the candidate gene approach and **(b) **the full-genome scan approach to finding susceptibility genes. Information from both approaches is used to guide the functional genomic study on multiple sets of microarray gene expression databases. This approach is powered by online statistical computation and a biomedical literature search.

Our idea is to turn to full genome functional studies for these goals. As illustrated in Figure 1, our approach takes advantage of the availability of abundant microarray data and a wealth of genomic/proteomic knowledge base from the public domain. Our intention is to integrate information from both the candidate gene and the full genome scan (thus far mostly family-based linkage) approaches. In this report we use two previously reported MS susceptibility genes, identified in the same study sample [7,9], namely *MBP *and *PRKCA*, as the lead to probe microarray gene expression data for functionally associated genes. High score genes, identified by statistical data analysis, are followed up by an extensive literature search for their biologic relevance.

Four large expression datasets are employed in this study (see Materials and methods, below). The first two, namely NCI_cDNA and NCI_Affy, are expression profiles for US National Cancer Institute (NCI)'s 60 human cancer cell lines reported by two different research teams [10,11]. The other two databases, GCN_2002 and GCN_2004, provide expression profiles for a diverse array of human tissues [12,13]. Together, they offer a glimpse into transcript regulation under a wide spectrum of physiologic conditions.

In addition to the conventional similarity study, we utilized a new computational tool, termed liquid association (LA) [14-16]. The power of the LA method in identifying elements of biologic pathways has been demonstrated by its use to identify correctly genes that are involved in the urea cycle [14]. In conventional similarity analysis, we tend to rely on the correlation corr(X,Y), which measures the degree of co-expression between two genes X and Y. Genes with high correlations are likely to be functionally associated. The encoded proteins may participate in the same pathway, form a common structural complex, or be regulated by the same mechanism. However, not all functionally associated genes are co-expressed; indeed, the majority of them are not. One conceivable reason for this is that gene expression can be sensitive to the often varying cellular state, such as presence or absence of hormones, metabolites, ion homeostasis, and so on. Two genes X and Y that are engaged in a common process under some conditions may disengage and embark on activities of their own as the cellular state changes. Consequently, two functionally related genes with a positive correlation in expression may become uncorrelated or even negatively correlated as the relevant state variable changes. If we could characterize the mediating state variable, then we might be able to detect the correlation by controlling the state variable.

**Figure 2:**
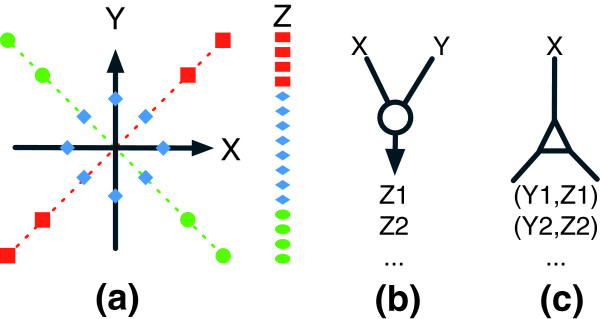
Liquid association. **(a) **Association between genes X and Y as mediated by gene Z. When gene Z is expressed at the high level (red), a positive correlation between X and Y is observed. The association changes as the expression of Z is lowered. It eventually becomes a negative trend (green). There are two basic ways (shown in panels b and c) to apply the liquid association (LA) scoring system to guide a genome-wide search. **(b) **When two genes X and Y are given, compute LA score *LA*(*X*, *Y*|*Z*) for every gene Z first and then output a short list of high score genes Z1, Z2, and so on. **(c) **When only one gene X is given, compute LA score *LA*(*X*, *Y*|*Z*) for every pair of genes X,Y first and then output a short list of high score gene pairs Y1,Z1, Y2,Z2, and so on.

Finding the mediating state variable is by no means simple. LA is a statistical device introduced for this purpose. The method is based on the assumption that the state variable is correlated with the expression of a third gene Z. If this is the case, then we may use Z to detect such a 'liquid' (as opposed to 'solid') pattern of statistical association between X and Y. Figure 2 illustrates how LA works. A liquid association score *LA*(*X*, *Y*|*Z*) can be computed using a simple statistical formula given in [14]. There are two ways of applying LA. For a given pair of X and Y, one can look for genes that may mediate X,Y co-expression by computing the LA score *LA*(*X*, *Y*|*Z*) for each gene Z in the genome and obtaining a genome-wide ranking. Alternatively, given one gene Z, we may ask which pairs of genes Z may mediate. With more computing effort, we can obtain *LA*(*X*, *Y*|*Z*) for every pair of genes X,Y and rank their scores in order to identify the most significant pairs. We have constructed a website to facilitate online searching for genes of interest. (See Additional data file 2 [Supplementary Text 1] for an illustrative application to the Alzheimer's hallmark gene *APP *[amyloid-β precursor protein].)

## Results

### *MBP*-initiated genome-wide liquid association search identifies *A2M*

We started with the *MBP *gene (which encodes myelin basic protein, an integral element of the myelin sheath surrounding the neuronal extensions). This gene is critical in triggering the immune reaction in the demyelination process for experimental allergic encephalomyelitis (EAE), a rodent model of MS. Importantly, *MBP *has been implicated both in linkage and in association studies conducted in MS pedigrees of Scandinavian origin (see Haines and coworkers [4] for references).

We applied the second genome-wide LA search method (Figure 2c) to the NCI_cDNA database. By treating *MBP *as the query gene X, we evaluate the LA score for every pair of genes (Y,Z). Because there are 9,076 genes in this database, about 49 million LA scores are computed and compared with each other. The output of a short list of 25 gene pairs with the best LA scores each from the positive and the negative ends is given in Additional data file 1 (Table S1). The statistical significance of the results of this gene search procedure is discussed in Additional data file 2 (Supplementary Text 3). We find that the gene *A2M *(encoding α_2_-macroglobulin, a cytokine transporter and protease inhibitor) appears many times. We further find an interesting biologic functional association between *A2M *and *MBP *from some literature about the pathogenesis of MS. Following demyelination in human MS and rodent EAE, immunogenic *MBP *peptides are released into cerebrospinal fluid and serum (see Oksenberg and coworkers [2] for references) and *A2M *represents the major MBP-binding protein in human plasma [17]. A significant increase in α_2_-macroglobulin is found in plasma of MS patients [18]. Analogously, in rodent EAE, infusion of α_2_-macroglobulin significantly reduces disease symptoms [19].

Among the genes to which *A2M *is paired, three are found to have functional association with immunologic neurodegenerative diseases. *LYST *(lysosomal trafficking regulator, also known as *CHS1*) is the causal gene for Chediak-Higashi syndrome, an inherited immunodeficiency disease, and *CHM *(Rab escort protein 1) is responsible for an inherited human retinal blindness known as choroideremia. (For details, see Online Mendelian Inheritance in Man of the National Center for Biotechnology Information [20].) *TRIB2 *(tribbles homolog 2) was identified as an autoantigen in autoimmune uveitis, a term encompassing a group of ocular inflammatory disorders with unknown causes [21]. Additionally, *MPDZ *(multiple PDZ domain protein) encodes a tight junction protein that is detected in noncompact regions of myelin, and it is thought to be required to maintain the cytoarchitecture of myelinating Schwann cells [22]. The biologic connection for other genes, many still of unknown function, is not clear. We compute the correlation between these genes and find that most of them have significant correlations. (See in Additional data file 2 [Supplementary Text 4] for more discussion.)

### Four multiple sclerosis loci from the Finnish population and *PRKCA*

Four major loci linked to MS have been identified in Finnish families: *HLA *on 6p21, *MBP *on 18q, and loci on 17q22-24 and 5p14-p12 [23]. These loci have also been implicated in other MS study samples from more heterogeneous populations [24,25]. The large locus on 17q was further refined to a 3 megabase (Mb) region in the Finnish MS families [23]. However, little information is available in the literature concerning how various loci are related to each other biologically. Most recently, association of specific *PRKCA *alleles at 17q24 with MS both in Finnish and Canadian MS study samples has been reported [7]. Involvement of *PRKCA *in MS was also validated by an association reported in a UK population [26]. *PRKCA *encodes a regulator of immune response, making it a highly suitable candidate gene for MS. A potential functional link between the *MBP *and *PRKCA *genes was identified by Feng and coworkers [27], who showed that a golli product of the myelin basic protein gene (*MBP*) can serve as a negative regulator of signaling pathways in T lymphocytes, particularly the protein kinase C pathway.

### *MBP-PRKCA*-initiated liquid association search identifies *SLC1A3*

To study the co-expression pattern between *MBP *and *PRKCA*, we took them as genes X and Y to explore the GNF_2002 database using our system. The gene with the greatest LA score was the gene *SLC1A3 *(glial high affinity glutamate transporter, member 3; see Additional data file 1 [Table S2]). Interestingly, *SLC1A3 *is located on 5p13.2 (36.6 to 36.7 Mb), within the previously identified MS locus on 5p [28], which is syntenic to the *EAE2 *locus in mouse.

### Test of the genetic relevance of *SLC1A3 *to multiple sclerosis

We wished to test whether there is any genetic relevance of *SLC1A3 *to MS. We selected five single nucleotide polymorphisms (SNPs) flanking the *SLC1A3 *gene (Table 1) to be genotyped in our primary study set, consisting of 61 MS families from the high-risk region of Finland. The most 5' SNP, namely rs2562582, located within 2 kilobases from the initiation of the *SLC1A3 *transcript, exhibited initial evidence for association with MS (*P *= 0.005) in the transmission disequilibrium test (TDT) analysis, suggesting a possible functional role for this variant in the transcriptional regulation of this gene. Moreover, as shown in Table 1, stratification of the Finnish MS families according to HLA genotype (using the SNP rs2239802, which exhibited strongest evidence for association in the Finnish families in the report by Riise Stensland and coworkers [28]), strengthened the association between the *SLC1A3 *SNP and MS (*P *= 0.0002, TDT). Thus, based on LA, and supported by association analyses in an MS study sample, the presence of *SLC1A3 *serves as a potential candidate to connect all four major MS loci identified in Finnish families, elucidating a potential functional relationship between genetically identified genes and loci. We consider further evidence in the following discussion.

**Table 1:** Genetic association results for SNPs located in the *SLC1A3 *gene in Finnish multiple sclerosis families

All families (n=69) and HLA stratified (n=38)				
SNP ID	TDT, *P* value	TDT, *P* value	MAF (CEPH)	MAF (Finnish)

rs2562582	0.005	0.0002	0.175	0.169
rs10941306	0.344	0.5	0.317	0.378
rs1366632	0.49	0.4898	0.458	0.459
rs1544795	0.477	0.5	0.467	0.437
rs1549627	0.5	0.4765	0.44	0.45

MAF (CEPH) is the minor allele frequency, as calculated from genotyping 30 trios belonging to Centre d'Etude du Polymorphisme Humain (CEPH) panel by HapMap project. MAF (Finnish) is the minor allele frequency calculated from genotyping the Finnish families used in this study. The transmission disequilibrium tests (TDTs) were calculated using ANALYZE software package [48]. In all, 69 families are included in this analysis, of which 38 are HLA stratified (multiple sclerosis families in which the affected individual had one or two HLA single nucleotide polymorphism [SNP] 2239802 risk alleles).

### Further liquid association analyses

**Figure 3:**
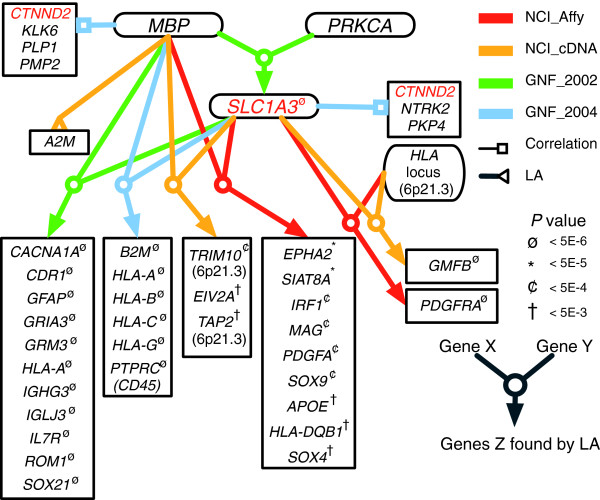
*SLC1A3 *and related genes. Four large-scale gene expression databases are used in this study. The arrows point to the genes found using the liquid association score system, according to the search method described in Figure 2b. The color of a line/arrow shows which database is used in the analysis. *P *values are calculated by randomization test. For descriptions of gene symbols, see Table 2. All four major multiple sclerosis loci for the Finnish scan have representative genes in this chart: *MBP *from 18q23, *PRKCA *from 17q22-q23.2, *SLC1A3 *from 5p13, and the *HLA *locus at 6p21.3. Also shown are two separate lists of genes correlated with *MBP *and with *SLC1A3 *most strongly. *CTNND2 *(located at 5p15.2) is seen in both lists.

**Figure 4:**
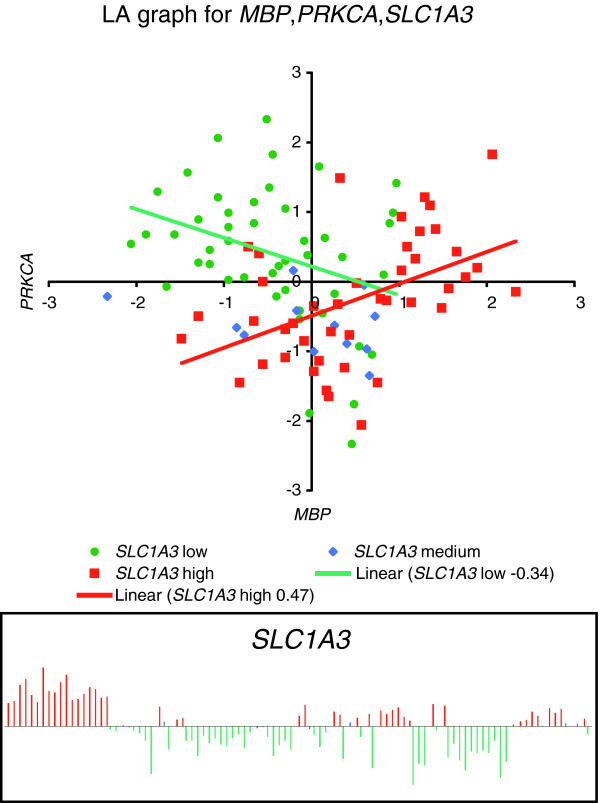
Liquid association activity plot for *MBP*, *PRKCA *as mediated by *SLC1A3*. When *SLC1A3 *is upregulated (red squares), a positive association between *MBP *and *PRKCA *can be seen. The correlation vanishes when the expression of *SLC1A3 *is low (green dots). Liquid association measures the change in the correlation structure; the score is 0.438 for this triplet.

We next took *MBP *and *SLC1A3 *as the query genes to conduct a genome-wide LA search in all four gene expression databases. Figure 3 and Table 2 highlight a set of genes whose biologic functions are most relevant to our MS study according to the literature. The detail LA outputs are given in Additional data file 1 (Tables S3 to S6). All LA plots are easy to generate online using our website. The one for the triplet including *MBP*, *PRKCA*, and *SLC1A3 *is shown in Figure 4.

**Table 2:** Genes detected in the liquid association analysis shown in Figure 1

Gene ID	Location	Description
*MBP*	18q23*	Myelin basic protein
*PRKCA*	17q24.2*	Protein kinase C, alpha
*SLC1A3*	5p13.2†	Solute carrier family 1 (glial high affinity glutamate transporter), member 3
*A2M*	12p13.31*	α_2_-Macroglobulin
*GRM3*	7q21.12	Glutamate receptor, metabotropic 3
*GFAP*	17q21.31	Glial fibrillary acidic protein
*CDR1*	Xq27.1	Cerebellar degeneration-related protein 1, 34kDa
*ROM1*	11q12.3	Retinal outer segment membrane protein 1
*CACNA1A*	19p13.2	Calcium channel, voltage-dependent, P/Q type, alpha 1A subunit
*GRIA3*	Xq25	Glutamate receptor, ionotrophic, AMPA3
*SOX21*	13q32.1	SRY (sex determining region Y)-box 21
*IL7R*	5p13.2†	Interleukin 7 receptor
*IGHG3*	14q32.33*	Immunoglobulin heavy constant gamma 3 (G3m marker)
*IGLJ3*	22q11.1	Immunoglobulin lambda joining 3
*HLA-A*	6p21.33†	Major histocompatibility complex, class I, A
*HLA-B*	6p21.33†	Major histocompatibility complex, class I, B
*HLA-C*	6p21.33†	Major histocompatibility complex, class I, C
*HLA-G*	6p22.1†	HLA-G histocompatibility antigen, class I, G
*B2M*	15q21.1	β_2_-Microglobulin
*PTPRC*	1q31.3*	Protein tyrosine phosphatase, receptor type, C
*EVI2A*	17q11.2*	Ecotropic viral integration site 2A
*TAP2*	6p21.32*	Transporter 2, ATP-binding cassette, sub-family B (MDR/TAP)
*TRIM10*	6p21.33†	Tripartite motif-containing 10
*SIAT1*	3q27.3	Sialyltransferase 1 (beta-galactoside alpha-2,6-sialyltransferase)
*MAG*	19q13.12*	Myelin associated glycoprotein [M29273]
*IRF1*	5q23.3*	Interferon regulatory factor 1
*APOE*	19q13.31*	Apolipoprotein E
*PDGFA*	7p22*	Platelet-derived growth factor-α polypeptide [H89357]
*SIAT8A*	12p12.1	Sialyltransferase 8A
*SOX4*	6p22.3†	SRY (sex determining region Y)-box 4
*SOX9*	17q24.3†	SRY (sex determining region Y)-box 9
*HLA-DQB1*	6p21.32†	Major histocompatibility complex, class II, DQ beta 1
*EPHA2*	1p36.13	EphA2 [T74614]
*GMFB*	14q22.2	Glia maturation factor, beta
*PDGFRA*	4q12*	Platelet-derived growth factor receptor-α polypeptide [M21574]
*PLP1*	Xq22.2*	Proteolipid protein 1
*KLK6*	19q13.41	Kallikrein 6 (neurosin, zyme)
*PMP2*	8q21.13	Peripheral myelin protein 2
*CTNND2*	5p15.2*	Catenin delta 2 (neural plakophilin-related arm-repeat protein)
*NTRK2*	9q21.33	Neurotrophic tyrosine kinase, receptor type 2
*PKP4*	2q24.1	Plakophilin 4

*Genes previously reported associated with MS. †Genes located within the previously reported MS susceptibility loci.

For GNF_2002 data, the gene with greatest LA score is *GRM3 *(glutamate receptor, metabotropic 3), followed by several genes involved in nervous diseases and neural development/functioning: *GFAP*, *CDR1*, *ROM1*, *CACNA1A*, and *GRIA3*. We also find *IL7R*, *IGHG3*, *IGLJ3*, and *HLA-A *among the highest scoring genes in this query. The identification of the *IL7R *by the LA analysis is particularly interesting because this gene was found to be associated with MS in the recent large international Whole Genome Association study [8].

### *MBP-SLC1A3 *initiated LA search identifies the HLA locus on 6p21

The locus of *HLA *on 6p21 is the only consensus MS locus replicated by genetic studies across different populations. Importantly, in the recent fine mapping effort with 1,068 SNPs covering the HLA locus and providing the SNP density of 1 SNP per 2 kilobases in the study sample of 4,200 individuals from Finnish and Canadian MS families [29], susceptibility to MS proved to be determined by *HLA-DRB1 *alleles and their interactions. Therefore, it is especially interesting that for the GNF_2004 data, eight of the 25 genes with the best LA scores are from the HLA locus: *HLA-A *(twice), *HLA-B *(twice), *HLA-C *(twice), and *HLA-G *(twice). Other HLA genes with very high LA scores include *HLA-E*, *HLA-F*, *HLA-DRA*, and *HLA-DPB1*. We also find *B2M *(which encodes β_2_-microglobulin, the light chain of MHC class I antigen) and a MS susceptibility gene, namely *CD45 *(a T-cell receptor for galectin-1).

### Additional functionally associated genes detected

The LA lists from NCI_cDNA data and NCI_Affy data also yielded several highly relevant candidate genes for MS, such as *MAG*, *IRF1*, *APOE*, *EIV2A*, and *PDGFA*. Also of interest are *SIAT8A*, *SIAT1*, *SOX4*, *SOX9*, and *EPHA2*. The protein encoded by *MAG *(myelin-associated glycoprotein) is involved in the process of myelination [30] and binds to sialic acid. *SIAT1 *and *SIAT8A *are both sialyltransferases. *SOX4 *and *SOX9 *are involved in central nervous system development [31,32]. *SOX4 *is required for the development of lymphocytes and thymocytes [33].

Results from the two NCI datasets also contain genes from the 6p21.3 locus: *TAP2*, *TRIM10*, and *HLA-DQB1*. A further investigation into the expressional association of the HLA family with *SLC1A3 *using the LA method finds two highly significant genes, namely *GMFB *and *PDGFRA *(see Additional data File 1 [Tables S7 and S8]). Also, these genes are be biologically relevant. *GMFB *(which encodes glia maturation factor beta) is reported to increase in astrocytes around the lesioned area after cortical cryogenic brain injury [34]. *PDGFRA *(the gene encoding platelet-derived growth factor receptor-α) is a well known marker for remyelination. The *PDGFA *supply may control oligodendrocyte progenitor cell numbers in the adult central nervous system as well as during development [35]. Interestingly, *CTNND2 *(catenin delta 2; neural plakophilin-related arm-repeat protein) is the fifth most correlated gene for *SLC1A3 *in the GNF_2004 data (see Additional data file 1 [Table S9]). It is also highly correlated with *MBP *(see Additional data file 1 [Table S10]).

## Discussion

We here introduce a novel bio-computational approach to identifying new candidate genes for genetic and functional studies of complex human traits. The initial result from the MS study is encouraging. We demonstrated that using only two genes, *MBP *and *PRKCA*, as the lead to probe for functionally associated genes, the LA method was successful in identifying a number of potential MS-related genes through subtle transcription co-regulation under a wide spectrum of cellular conditions. Additionally, when *MBP *and *SLC1A3 *were used as query genes in the LA analysis, the recently identified MS susceptibility gene *IL7R *was among the highest scoring, statistically significant genes.

LA allows the detection of gene co-regulation, which may only occur under specific cellular states. There is no need to specify the states, and this is one of the advantages of LA [14]. Furthermore, LA can be used in conjunction with traditional correlation analysis. An online computation system to conduct both LA and correlation analysis is available at our website [36]. This platform allows users to switch conveniently from one gene expression dataset to another. Those conducting research in other diseases can easily carry out analyses similar to that presented here with a few leading genes related to the disease of interest.

### Glutamate-induced excitotoxicity

Although all of the putative genes identified using LA method must be confirmed with genetic association studies in multiple populations and eventually in targeted functional studies, the putative genes identified here are highly relevant to MS. Our transcript regulatory findings portray a coherent web of molecular evidence, which supports the glutamate-induced excitotoxicity hypothesis of MS. *SLC1A3 *is highly expressed in various brain regions including cerebellum, frontal cortex, basal ganglia, and hippocampus. It encodes a sodium-dependent glutamate/aspartate transporter 1 (GLAST). Glutamate and aspartate are excitatory neurotransmitters that have been implicated in a number of pathologies the nervous system. Glutamate concentration in cerebrospinal fluid rises in acute MS patients [37], whereas the glutamate antagonist amantadine reduces MS relapse rate [38]. In EAE, the levels of GLAST and GLT-1 (*SLC1A2*) have been reported to be downregulated in spinal cord at the peak of disease symptoms, and no recovery was observed after remission [39]. We consider it encouraging that several lines of evidence, including both genetic association and gene expression association, are consistent with the glutamate-induced excitotoxicity hypothesis, which states that glutamate-induced excitotoxicity results in demyelination and axonal damage in MS [40].

### International multiple sclerosis Whole Genome Association study

The recent international MS Whole Genome Association scan [8] provided additional evidence supporting an association between MS susceptibility and *SLC1A3*. A major component of the study is the use of Affymetrix 500K to screen common genetic variants of 931 family trios. Using the online supplementary information provided by the International MS Genetics Consortium [8] we found two SNPs, namely rs4869676 (chromosome 5: 36641766) and rs4869675 (chromosome 5: 36636676), with TDT *P *values of 0.0221 and 0.00399, respectively, which are in the upstream regulatory region of the *SLC1A3 *gene. In fact, within the 1 Mb region of rs486975 there are a total 206 SNPs in the Affymetrix 500K chip. No other SNPs have *P *values less than that of rs486975. The next most significant SNPs in this region are rs1343692(chromosome 5: 35860930) and rs6897932 (chromosome 5: 35910332; the identified MS susceptibility SNP in the *IL7R *axon). The MS marker we identified, rs2562582 (chromosome 5: 36641117), less than 5 kilobases away from rs4869675, was not used in the Affymetrix chip.

Although the results reported here should be considered preliminary, we propose that the genes and networks identified should be targets for additional analyses of MS in different study populations.

### Use of public gene expression data

One unique feature of our approach to finding candidate genes is the use of public domain gene expression databases, of which the original experiments were not designed to study our disease of interest. For example, the two NCI-60 cell line (a panel of 60 diverse human cancer cell lines) gene expression data have primarily been used to aid anticancer drug screening, not for the study of MS. With our promising initial findings, we expect our functional genomics approach to be applicable in the initial identification of involved molecular pathways in the pathogenesis of other complex diseases. Investigators may apply our LA method or bring in other computational methods to data mine the numerous free public gene expression databases, thus reducing the time and expense associated with disease gene identification.

## Materials and methods

### Gene expression datasets

Four large-scale gene expression databases are employed in this study, with various numbers of conditions and genes. The first two databases give expression profiles for the 60 representative cell lines from seven cancer types that have been used in NCI's anticancer drug screen. The NCI_cDNA database uses the cDNA microarray reported by investigators from P Brown's laboratory at Stanford University [10], whereas the NCI_Affy uses Affymetrix oligonucleotide high-density HU6800 arrays [11]. The two other databases [12,13] are samples from diverse array of human tissues. GNF_2002 has a probe set for a total of 12,533 genes/clones and 101 chips (using Affymetrix U95A arrays) and GNF_2004 has a probe set for 33,689 genes and 158 chips (using Affymetrix HG-U133A and GNF1H; data downloaded from the Gene Expression Atlas [41]). The corresponding numbers for NCI_Affy are 5,611 and 60 (data downloaded from the supplementary data file of Staunton and coworkers [42]), and for NCI_cDNA they are 9,703 and 60 (data downloaded from the NCI60 Cancer Microarray Project) [43].

### Liquid association

To compute the liquid association score *LA*(*X*, *Y*|*Z*) for a triplet of genes, normal score transformation is first applied for each gene. After transformation, *LA*(*X*, *Y*|*Z*) is given by the average of triple product between X, Y and Z: *LA*(*X*, *Y*|*Z*) = (*x*_1_*y*_1_*z*_1 _+ ... *x*_*m*_*y*_*m*_*z*_*m*_)/*m*. For a given pair (X,Y), the test of significance of an LA score is conducted by permutation, as previously described [14,16] and the *P *value is reported in each of our LA output tables. In addition, to help with the interpretation of the effect size of the LA score, two algorithms were used to find the correlation change between the state of high expression of the LA scouting gene and the state of low expression (see Additional data file 2 [Supplementary Text 5]). The LA website [36] was created to facilitate the online computation of LA. High score output genes are returned to user's browser for immediate connection to Entrez Gene. The website also generates LA graphs, performs standard correlation analysis, and provides summary information regarding gene location, functional annotation, and so on.

### Multiple sclerosis association study sample

The study set used for the association analysis contained 28 multiplex MS families with multiple affected individuals, and 41 nuclear MS families (MS patient and his/her parents and, in case of a missing parent, healthy siblings were included). Twenty-two of the 28 multiplex families and all trio families originated from Southern Ostrobothnia region of Finland, which has an especially high incidence and prevalence of MS. All families were Finnish and of Caucasian descent, and they have been described in more detail by Saarela and coworkers [23]. Diagnosis of MS in affected individuals strictly followed Poser's diagnostic criteria [44]. All individuals gave informed consent and the study was approved by the Ethics Committee for Ophthalmology, Otorhinolaryngology, Neurology, and Neurosurgery in the Hospital District of Helsinki and Uusimaa. (decision 46/2002, DNRO 192/E9/02).

### Genotyping

To control for sample mix-ups, all samples were genotyped for determining the sex and four microsatellite markers using the ABI 3730 (Applied Biosystems, Foster City, CA, USA). The data were compared with the known sex of the samples and checked for Mendelian errors. No Mendelian discrepancies were observed in this study set. To select the initial set of SNPs used for the association analysis, we set up the following criteria: each of the markers should be highly polymorphic in Centre d'Etude du Polymorphisme Humain (CEPH) reference families genotyped in the HapMap project and should belong to unique solid line linkage disequilibrium haplotype blocks as defined by Haploview's version 3.11 [45]. We found five highly polymorphic SNPs within and in the proximity of *SLC1A3 *gene that belonged to separate haplotype blocks, according to the HapMap data. The SNPs were genotyped using multiplexed allele-specific primer extension on microarrays [46]. Primers for multiplex polymerase chain reactions were designed using in-house scripts written for the Primer3 program [47], and an in-house built software package SNPSnapper, version 1.38beta, was utilized to call genotypes automatically.

### Statistical analyses for genotyping

Allele and genotype frequencies were determined from the data, and deviation from the Hardy-Weinberg equilibrium was tested using Pearson's χ^2 ^test. We used the ANALYZE package to conduct TDT analyses to test for association between MS and *SLC1A3 *gene [48].

## Abbreviations

CEPH, Centre d'Etude du Polymorphisme Humain; EAE, experimental allergic encephalomyelitis; GLAST, glutamate/aspartate transporter 1; HLA, human leukocyte antigen; LA, liquid association; Mb, megabase; MHC, major histocompatibility complex; MS, multiple sclerosis; NCI, National Cancer Institute; SNP, single nucleotide polymorphism; TDT, transmission disequilibrium test.

## Authors' contributions

SY and KCL contributed equally to statistical computing. DB, DC, JS, and OWC performed genotyping analysis. KCL, SY, and XW conducted LA analysis. KCL, AP, and LP designed the research and provided funding for research. KCL, AP, SY, DB, and LP wrote the paper.

## Additional data files

The following additional data are available with the online version of this paper. Additional data file 1 contains ten tables including results from both LA and correlation analyses. Additional data file 2 contains supplementary text detailing the additional data analyses mentioned in the text.

## Supplementary Material

Additional data file 1 Presented are ten tables including results from both LA and correlation analyses. Click here for file

Additional data file 2 Supplementary text detailing the additional data analyses mentioned in the text is provided. Click here for file
