# Supplementary material for: Finding disease candidate genes by liquid association
Source: Genome Biol. 2007 Oct 4;8(10):R205. doi: 10.1186/gb-2007-8-10-r205 (PMC2246280; doi:10.1186/gb-2007-8-10-r205)
Supplement: Additional data file 1 — Presented are ten tables including results from both LA and correlation analyses. [file gb-2007-8-10-r205-S1.pdf]

Table S.1: LA output for X=MBP Y=Any Z=Any (NCI_cDNA)

Y	Z	LAP	PVAL	CC (L)	CC (H)	CC (H-L)	Y	Z	LAP	PVAL	CC (L)	CC (H)	CC (H-L)
**A2M**	ZBTB40	0.7272	0	-0.78	0.95	1.73	FCGR2A	RNASEH2A	-0.6871	0	0.78	-0.62	-1.40
**A2M**	***MPDZ***	0.6937	0	-0.69	0.93	1.63	NA-3171	ITGA9	-0.6755	0	0.69	-0.88	-1.57
CYP3A7	ACLY	0.6608	0	-0.91	0.90	1.81	**A2M**	PPA1	-0.6483	0	0.75	-0.60	-1.35
**A2M**	AA040811	0.6579	0	-0.81	0.68	1.49	C10orf116	ENTPD5	-0.631	0	0.79	-0.53	-1.32
**A2M**	***TRIB2***	0.6506	0	-0.64	0.86	1.50	FCGR2A	OCIAD2	-0.6259	0	0.93	-0.81	-1.74
**A2M**	C2orf32	0.6311	0	-0.68	0.81	1.50	NA-4174	MRPL11	-0.625	0	0.92	-0.38	-1.30
**A2M**	CPEB2	0.6258	0	-0.65	0.76	1.41	NA-3171	W91930	-0.6245	0	0.81	-0.85	-1.66
**A2M**	N66535	0.624	0	-0.79	0.81	1.60	**A2M**	SNRPE	-0.6244	0	0.84	-0.82	-1.66
TMF1	NA-4174	0.6167	0	-0.54	0.84	1.38	OBSL1	DHPS	-0.6163	0	0.79	-0.51	-1.30
**A2M**	ZNF598	0.6164	0	-0.72	0.69	1.41	UBE2I	GSN	-0.6156	0	0.72	-0.93	-1.65
**A2M**	C11orf41	0.6084	0	-0.72	0.68	1.41	GINS1	SRF	-0.61	0	0.79	-0.58	-1.38
**A2M**	***LYST***	0.6057	0	-0.59	0.68	1.27	NA-4174	PPA1	-0.6079	0	0.85	-0.54	-1.39
**A2M**	KIAA1539	0.6017	0	-0.67	0.80	1.47	BACE2	SNRPD1	-0.6066	0	0.89	-0.17	-1.06
PPA1	MAP4	0.5987	0	-0.76	0.67	1.43	NA-4174	WDR74	-0.6058	0	0.92	-0.74	-1.66
GINS1	SORCS1	0.5979	1.0E-06	-0.44	0.72	1.15	FMO4	MRPL9	-0.6017	0	0.82	-0.53	-1.35
**A2M**	***CHM***	0.5935	0	-0.77	0.65	1.42	APH1A	PLSCR3	-0.5966	1.0E-06	0.81	-0.71	-1.52
T64854	BRPF1	0.5904	0	-0.39	0.80	1.19	C6orf206	CAMSAP1	-0.5912	0	0.87	-0.82	-1.69
RNASET2	ZNF488	0.5903	0	-0.73	0.65	1.37	PANX1	ZNF605	-0.5858	0	0.71	-0.88	-1.59
NA-3171	ACACA	0.5885	1.0E-06	-0.86	0.56	1.43	**A2M**	L3MBTL2	-0.5858	0	0.75	-0.66	-1.41
HSPC159	NA-562	0.5869	0	-0.31	0.77	1.08	GART	SNTA1	-0.5836	0	0.67	-0.77	-1.45
NA-562	MPDZ	0.5858	0	-0.50	0.64	1.14	CPB1	DR1	-0.5821	0	0.75	-0.73	-1.49
**A2M**	C1orf61	0.5856	0	-0.53	0.65	1.18	KATNA1	TM2D1	-0.5814	0	0.71	-0.57	-1.28
FLJ37953	ZBTB40	0.5846	0	-0.78	0.74	1.53	ACACA	RUFY3	-0.5813	0	0.68	-0.86	-1.55
**A2M**	LUZP1	0.5844	1.0E-06	-0.79	0.83	1.63	**A2M**	GRK6	-0.581	0	0.81	-0.69	-1.50
**A2M**	TMF1	0.5839	0	-0.57	0.78	1.36	APH1A	C14orf109	-0.581	1.0E-06	0.86	-0.66	-1.52

Table S.1 In this table, only columns for Y and Z are displayed. All triplets have the same X, ***MBP. A2M*** is found 15 times in top 25 triplets and 4 times in bottom 25 triplets when ordered by LA score. Annotations for genes highlighted in this table are ***MBP*** (myelin basic protein), ***A2M*** (alpha-2-macroglobulin), ***MPDZ*** (multiple PDZ domain protein), ***TRIB2*** (Tribbles homolog 2 (Drosophila)), ***LYST*** (lysosomal trafficking regulator), ***CHM*** (choroideremia (Rab escort protein 1)), ***ZBTB40*** (zinc finger and BTB domain containing 40), ***C2orf32*** (chromosome 2 open reading frame 32), ***CPEB2*** (cytoplasmic polyadenylation element binding protein 2), ***ZNF598*** (zinc finger protein 598), ***C11orf41*** (chromosome 11 open reading frame 41), ***C1orf61*** (chromosome 1 open reading frame 61), ***LUZP1*** (leucine zipper protein 1), ***TMF1*** (TATA element modulatory factor 1). The p-value is estimated by permuting Y and Z 1,000,000 times. CC(L) and CC(H) give the correlation between the LA pair when the expression level of the mediator gene is low (for L) and high (for H) respectively. CC(H-L) equals CC(H)-CC(L).

Table S.2: LA output for X=PRKCA Y=MBP Z=Any (GNF_2002)

Z	LAP	PVAL	CC (L)	CC (H)	CC (H-L)	Z	LAP	PVAL	CC (L)	CC (H)	CC (H-L)
**SLC1A3**	0.438	0	-0.34	0.49	0.84	RBM4	-0.3952	0	0.60	-0.50	-1.10
39765_at	0.413	0	-0.32	0.64	0.96	KNTC1	-0.3759	0	0.23	-0.59	-0.81
GATM	0.4123	0	-0.69	0.40	1.09	CHD1	-0.3738	0	0.37	-0.58	-0.95
FAM107A	0.3895	0	-0.46	0.25	0.70	FADD	-0.3681	0	0.50	-0.41	-0.92
37060_at	0.387	0	-0.36	0.63	0.99	SFRS3	-0.3623	1.0E-06	0.17	-0.56	-0.73
NTRK2	0.3859	0	-0.53	0.29	0.82	NUP160	-0.3524	1.0E-06	0.38	-0.54	-0.92
SNCB	0.3822	0	-0.44	0.74	1.18	PTBP1	-0.3522	2.0E-06	0.61	-0.57	-1.18
BCL2L2	0.3809	0	-0.76	0.44	1.19	DAP3	-0.3465	0	0.29	-0.64	-0.93
LOC201229	0.3781	0	-0.58	0.32	0.90	COPB1	-0.3454	1.0E-06	0.28	-0.58	-0.86
CLU	0.3705	0	-0.66	0.49	1.15	DDX46	-0.3452	1.0E-06	0.31	-0.58	-0.89
32345_at	0.3658	1.0E-06	-0.41	0.48	0.89	CASP8	-0.3445	1.0E-06	0.52	-0.72	-1.24
AMPH	0.3637	0	-0.31	0.69	1.00	ATP11B	-0.3444	0	0.29	-0.56	-0.85
PHLPP	0.3628	0	-0.58	0.10	0.68	NUP205	-0.3443	0	0.40	-0.52	-0.91
ATP1B2	0.3624	0	-0.49	0.44	0.93	1843_at	-0.343	0	0.27	-0.58	-0.85
STOML1	0.3595	0	-0.58	0.29	0.88	LMNB1	-0.3425	0	0.23	-0.57	-0.80
ZBTB16	0.358	1.0E-06	-0.64	0.30	0.94	ARIH1	-0.3417	1.0E-06	0.25	-0.57	-0.82
ABLIM3	0.3553	1.0E-06	-0.54	0.28	0.82	FAM32A	-0.3399	3.0E-06	0.50	-0.72	-1.21
THRA	0.3552	2.0E-06	-0.51	0.41	0.92	XRCC5	-0.3366	0	0.24	-0.55	-0.79
DNAJB2	0.3538	0	-0.62	0.08	0.70	CLIC1	-0.3363	1.0E-06	0.44	-0.59	-1.03
CRYAB	0.3537	2.0E-06	-0.59	0.44	1.03	SMARCC1	-0.335	0	0.32	-0.51	-0.83

Table S.2 In this table, only column Z is displayed. All triplets have the same X, ***PRKCA***, and Y, ***MBP. SLC1A3*** is found with highest positive LA score. Annotations for genes highlighted in this table are ***PRKCA*** (protein kinase C, alpha), ***MBP*** (myelin basic protein), ***SLC1A3*** (solute carrier family 1 (glial high affinity glutamate transporter), member 3). The p-value is estimated by permuting Z 1,000,000 times.

Table S.3: LA output for X=SLC1A3 Y=MBP Z=Any (GNF_2002)

Z	LAP	PVAL	CC (L)	CC (H)	CC (H-L)	Z	LAP	PVAL	CC (L)	CC (H)	CC (H-L)
**GRM3**	0.6359	0	-0.08	0.83	0.91	TPT1	-0.6312	0	0.82	0.02	-0.80
**CDR1**	0.6027	0	-0.10	0.84	0.94	POLD4	-0.5502	0	0.84	0.13	-0.71
**ROM1**	0.5947	0	-0.03	0.77	0.80	S100A2	-0.5436	0	0.79	0.08	-0.71
TYRO3	0.5946	0	-0.27	0.81	1.08	NUBP1	-0.5352	0	0.84	0.03	-0.81
**GFAP**	0.5929	0	-0.14	0.81	0.95	B4GALT1	-0.532	0	0.83	0.10	-0.73
TRIM9	0.5914	0	0.11	0.80	0.69	ETV6	-0.5308	0	0.76	0.22	-0.54
ABCA2	0.5814	0	0.02	0.81	0.79	RPS27	-0.5286	0	0.77	0.29	-0.48
B3GNT1	0.5743	0	-0.54	0.80	1.34	KLF11	-0.5279	0	0.76	0.10	-0.67
**SOX21**	0.5743	0	0.17	0.81	0.64	STAT6	-0.5237	0	0.79	0.18	-0.60
HIPK2	0.5741	0	0.14	0.85	0.71	**IGHG3**	-0.523	0	0.74	0.22	-0.52
**CACNA1A**	0.5707	0	0.10	0.86	0.76	**IGLJ3**	-0.5216	0	0.86	0.14	-0.72
PALM	0.5668	0	-0.20	0.78	0.98	SLC24A1	-0.5214	0	0.76	-0.03	-0.78
**GRIA3**	0.5668	0	-0.07	0.80	0.87	XBP1	-0.5191	0	0.79	-0.05	-0.84
SLCO3A1	0.5644	0	-0.12	0.70	0.81	**IL7R**	-0.5179	0	0.79	0.07	-0.72
KIAA0523	0.5638	0	0.05	0.83	0.78	**HLA-G**	-0.5128	0	0.78	0.01	-0.77
SYNJ2	0.5611	0	0.13	0.77	0.64	STATH	-0.5123	0	0.74	0.23	-0.51
MAPT	0.5591	0	0.13	0.77	0.64	CFD	-0.5119	0	0.80	0.03	-0.77
MRAS	0.559	0	0.13	0.77	0.65	MBOAT5	-0.5116	0	0.76	0.23	-0.53
DCTN1	0.5583	0	-0.67	0.81	1.48	LYN	-0.5083	0	0.75	0.14	-0.61
BTBD3	0.558	0	0.12	0.77	0.65	MYOC	-0.5064	0	0.80	0.00	-0.80

Table S.3 In this table, only column Z is displayed. All triplets have the same X, ***SLC1A3***, and Y, ***MBP.*** Annotations for highlighted genes in this table are ***MBP*** (myelin basic protein), ***SLC1A3*** (solute carrier family 1 (glial high affinity glutamate transporter), member 3), ***GRM3*** (glutamate receptor, metabotropic 3), ***GFAP*** (glial fibrillary acidic protein), ***CDR1*** (cerebellar degeneration-related protein 1, 34kDa), ***ROM1*** (retinal outer segment membrane protein 1), ***CACNA1A*** (calcium channel, voltage-dependent, P/Q type, alpha 1A subunit), ***GRIA3*** (glutamate receptor, ionotrophic, AMPA 3), ***SOX21*** (SRY (sex determining region Y)-box 21), ***IL7R*** (interleukin 7 receptor), ***IGHG3*** (immunoglobulin heavy constant gamma 3 (G3m marker)), ***IGLJ3*** (immunoglobulin lambda joining 3), ***HLA-G*** (major histocompatibility complex, class I, G). The p-value is estimated by permuting Z 1,000,000 times.

Table S.4: LA output for X=SLC1A3 Y=MBP Z=Any (GNF_2004)

Z	LAP	PVAL	CC (L)	CC (H)	CC (H-L)	Z	LAP	PVAL	CC (L)	CC (H)	CC (H-L)
KIAA1463	0.5828	0	-0.11	0.75	0.86	PNRC1	-0.5585	0	0.77	-0.66	-1.44
TTYH2	0.569	0	-0.05	0.76	0.81	Hs.390440	-0.5449	0	0.68	-0.59	-1.27
C3orf4	0.5636	0	0.08	0.80	0.71	CD48	-0.5411	0	0.74	-0.53	-1.27
SLC9A6	0.5603	0	-0.05	0.70	0.76	**PTPRC**	-0.5372	0	0.79	-0.34	-1.13
AHCYL1	0.5524	0	-0.06	0.66	0.72	ARF6	-0.535	0	0.63	-0.52	-1.15
AKAP11	0.5514	0	-0.10	0.79	0.88	TINF2	-0.5346	0	0.58	-0.50	-1.08
QKI	0.551	0	-0.21	0.74	0.95	LITAF	-0.5343	0	0.54	-0.39	-0.93
SKP1A	0.5508	0	0.12	0.70	0.58	**HLA-G**	-0.5323	0	0.64	-0.58	-1.22
FAIM2	0.5479	0	-0.19	0.73	0.92	**B2M**	-0.5301	0	0.63	-0.46	-1.09
C3orf4	0.5465	0	-0.11	0.70	0.81	DSIPI	-0.5281	0	0.54	-0.53	-1.07
KIF1B	0.5455	0	0.06	0.74	0.68	**B2M**	-0.5261	0	0.72	-0.44	-1.16
SCRN1	0.5432	0	0.01	0.77	0.76	STAT6	-0.5256	0	0.70	-0.49	-1.19
PEA15	0.5415	0	-0.14	0.75	0.89	**HLA-B**	-0.5187	0	0.71	-0.60	-1.31
SAP18	0.5411	0	-0.16	0.76	0.91	CMRF-35H	-0.5172	0	0.56	-0.62	-1.18
PAFAH1B1	0.5367	0	0.15	0.78	0.63	**HLA-A**	-0.5172	0	0.64	-0.43	-1.07
KIAA1463	0.5367	0	-0.05	0.64	0.69	CFLAR	-0.5168	0	0.66	-0.36	-1.02
GDI1	0.5351	0	-0.04	0.75	0.78	LITAF	-0.5163	0	0.67	-0.49	-1.16
LANCL1	0.5342	0	0.10	0.70	0.60	**HLA-G**	-0.5163	0	0.67	-0.61	-1.28
TRIM37	0.5335	0	0.07	0.73	0.65	**HLA-A**	-0.5161	0	0.70	-0.43	-1.13
RTN3	0.5334	0	-0.19	0.68	0.87	**HLA-C**	-0.5142	0	0.63	-0.49	-1.12
DCTN1	0.5331	0	-0.22	0.63	0.86	KIAA0247	-0.512	0	0.73	-0.62	-1.35
KIAA1189	0.5325	0	-0.33	0.58	0.91	**HLA-C**	-0.5118	0	0.68	-0.63	-1.31
CDW92	0.5325	0	-0.06	0.78	0.85	TAPBPL	-0.5107	0	0.63	-0.55	-1.18
C11orf9	0.5325	0	-0.11	0.75	0.86	**HLA-B**	-0.5105	0	0.65	-0.62	-1.27
KIF1B	0.5301	0	-0.05	0.71	0.76	FLJ21438	-0.5105	0	0.63	-0.37	-1.00

Table S.4 In this table, only column Z is displayed. All triplets have the same X, ***SLC1A3***, and Y, ***MBP.*** Annotations for highlighted genes in this table are ***MBP*** (myelin basic protein), ***SLC1A3*** (solute carrier family 1 (glial high affinity glutamate transporter), member 3), ***HLA-A*** (major histocompatibility complex, class I, A), ***HLA-B*** (major histocompatibility complex, class I, B), ***HLA-G*** (HLA-G histocompatibility antigen, class I, G), ***HLA-C*** (major histocompatibility complex, class I, C), ***B2M*** (beta-2-microglobulin), ***PTPRC*** (protein tyrosine phosphatase, receptor type, C). The p-value is estimated by permuting Z 1,000,000 times.

Table S.5: LA output X=SLC1A3 Y=MBP Z=Any (NCI_Affy)

Z	LAP	PVAL	CC (L)	CC (H)	CC (H-L)	Z	LAP	PVAL	CC (L)	CC (H)	CC (H-L)
HOXA5	0.514	1.2E-05	-0.69	0.74	1.43	NR4A1	-0.4575	4.2E-05	0.60	-0.48	-1.08
CALB2	0.487	1.4E-05	-0.55	0.80	1.35	**ST8SIA1**	-0.4552	3.5E-05	0.68	-0.81	-1.49
TFAM	0.4816	4.0E-06	-0.70	0.43	1.13	CYP2C9	-0.4523	5.3E-05	0.64	-0.45	-1.09
AKR1D1	0.4764	5.8E-05	-0.54	0.78	1.33	**ST8SIA1**	-0.4496	6.5E-05	0.73	-0.83	-1.56
LPL	0.466	8.9E-05	-0.50	0.73	1.23	U06715	-0.4486	1.8E-05	0.38	-0.67	-1.05
PTK2B	0.4645	8.7E-05	-0.75	0.61	1.36	PCSK6	-0.4453	2.7E-05	0.60	-0.86	-1.47
**EPHA2**	0.4579	3.9E-05	-0.51	0.67	1.18	FXYD1	-0.4418	2.0E-04	0.58	-0.65	-1.23
**EPHA2**	0.4497	6.4E-05	-0.56	0.69	1.25	IGF1	-0.435	2.5E-04	0.37	-0.61	-0.99
DNM1	0.4485	6.7E-05	-0.59	0.60	1.19	GPR143	-0.4311	9.2E-05	0.52	-0.64	-1.17
DAP	0.4475	6.0E-05	-0.55	0.42	0.98	**ST8SIA1**	-0.429	3.0E-04	0.57	-0.67	-1.24
KDELR2	0.4458	6.8E-05	-0.66	0.64	1.30	HTR2B	-0.428	9.6E-05	0.39	-0.61	-1.00
H89357	0.4376	1.0E-04	-0.58	0.69	1.27	EGR3	-0.4235	3.2E-04	0.60	-0.54	-1.14
SHROOM3	0.4342	1.1E-04	-0.68	0.41	1.09	GYPC	-0.417	2.0E-04	0.60	-0.45	-1.05
**EPHA2**	0.4322	1.0E-04	-0.54	0.55	1.09	RUNX1	-0.417	1.4E-04	0.46	-0.47	-0.93
GABRR2	0.4306	2.4E-04	-0.73	0.56	1.28	SLC25A4	-0.4143	8.2E-05	0.71	-0.64	-1.34
F13B	0.4285	3.1E-04	-0.45	0.60	1.06	BMP7	-0.4074	2.7E-04	0.63	-0.67	-1.30
CANX	0.4228	3.8E-04	-0.43	0.63	1.07	SREBF2	-0.4058	1.2E-04	0.37	-0.69	-1.06
HOXA5	0.4225	1.1E-04	-0.59	0.70	1.29	CYP2C9	-0.4007	7.6E-04	0.54	-0.38	-0.92
**EPHA2**	0.4222	4.0E-04	-0.52	0.61	1.13	SAT1	-0.3986	1.6E-04	0.46	-0.67	-1.12
HOXA5	0.422	1.7E-04	-0.53	0.52	1.05	ITGB3	-0.3975	2.6E-04	0.48	-0.74	-1.22
CHGB	0.4217	4.0E-04	-0.77	0.77	1.54	**MAG**	-0.3969	4.4E-04	0.76	-0.73	-1.49
IL15	0.4207	1.8E-04	-0.47	0.55	1.02	AP1B1	-0.3948	1.7E-04	0.45	-0.68	-1.13
CSNK1A1	0.4206	1.9E-04	-0.71	0.72	1.43	CLCN6	-0.3938	3.1E-04	0.45	-0.72	-1.17
RIT1	0.4168	2.0E-04	-0.57	0.79	1.36	GYPC	-0.3938	1.0E-03	0.44	-0.50	-0.94
DSG3	0.415	2.3E-04	-0.55	0.56	1.11	CAPN3	-0.3937	4.3E-04	0.56	-0.34	-0.90
**EPHA2**	0.4149	2.0E-04	-0.62	0.67	1.29	IFNA5	-0.3888	1.1E-03	0.48	-0.76	-1.24
YY1	0.4138	2.3E-04	-0.50	0.47	0.97	FOLR2	-0.3888	5.2E-04	0.70	-0.85	-1.54
SEMA3F	0.4138	2.4E-04	-0.57	0.91	1.48	MYL3	-0.3881	5.7E-04	0.87	-0.56	-1.42
TGFA	0.4136	5.0E-04	-0.57	0.57	1.14	EGR3	-0.3875	4.0E-04	0.48	-0.36	-0.84
CALB2	0.4134	5.1E-04	-0.51	0.42	0.93	CHRNA7	-0.3851	1.2E-03	0.53	-0.53	-1.06

Z	LAP	PVAL	CC (L)	CC (H)	CC (H-L)	Z	LAP	PVAL	CC (L)	CC (H)	CC (H-L)
**IRF1**	0.4134	1.7E-04	-0.77	0.75	1.52	SEPT2	-0.3844	3.2E-04	0.71	-0.61	-1.33
ZFP36	0.41	3.0E-04	-0.88	0.67	1.55	AP1B1	-0.3831	3.0E-04	0.71	-0.70	-1.41
DNM1	0.4096	2.0E-04	-0.57	0.53	1.10	**APOE**	-0.3829	7.2E-04	0.46	-0.61	-1.07
GPX2	0.4094	2.9E-04	-0.64	0.61	1.25	PTPN1	-0.3825	7.0E-04	0.43	-0.57	-1.00
L14837	0.4091	2.8E-04	-0.59	0.48	1.07	CD63	-0.3817	3.2E-04	0.44	-0.60	-1.04
MAT1A	0.4071	2.8E-04	-0.53	0.44	0.97	**MAG**	-0.3814	1.3E-03	0.69	-0.74	-1.43
FLT3	0.4057	6.7E-04	-0.51	0.62	1.14	GYG2	-0.3809	5.5E-04	0.75	-0.81	-1.56
LPL	0.4043	3.3E-04	-0.55	0.35	0.90	MERTK	-0.3794	3.3E-04	0.59	-0.64	-1.23
BCL7B	0.4032	7.2E-04	-0.64	0.72	1.35	MBP	-0.3789	1.5E-03	0.59	-0.47	-1.06
H64564	0.4016	3.4E-04	-0.61	0.42	1.03	AP1B1	-0.3773	4.0E-04	0.82	-0.71	-1.53
RBBP5	0.4011	3.6E-04	-0.57	0.84	1.41	**HLA-DQB1**	-0.3768	1.5E-03	0.46	-0.52	-0.97
STT3A	0.4011	3.6E-04	-0.71	0.44	1.15	F5	-0.3764	4.2E-04	0.34	-0.57	-0.90
CPB2	0.4008	3.4E-04	-0.54	0.72	1.26	MXI1	-0.375	4.3E-04	0.63	-0.64	-1.27
CFB	0.4001	3.5E-04	-0.63	0.54	1.17	MKRN3	-0.3748	8.2E-04	0.59	-0.56	-1.15
**SOX9**	0.3989	3.9E-04	-0.59	0.80	1.39	ZRANB2	-0.3747	1.6E-03	0.48	-0.43	-0.92
CRAT	0.3987	4.0E-04	-0.44	0.83	1.28	PTPN1	-0.3745	4.2E-04	0.43	-0.73	-1.16
**SOX9**	0.3982	7.7E-04	-0.70	0.71	1.41	NRCAM	-0.3742	4.2E-04	0.29	-0.73	-1.02
BCL7B	0.3978	3.8E-04	-0.26	0.68	0.95	ELK4	-0.3737	9.0E-04	0.56	-0.38	-0.95
M24594	0.3974	4.3E-04	-0.74	0.71	1.45	T75577	-0.3736	4.1E-04	0.23	-0.68	-0.92
ISGF3G	0.3973	4.0E-04	-0.60	0.37	0.97	**SOX4**	-0.373	9.8E-04	0.52	-0.38	-0.90

Table S.5 In this table, only column Z is displayed. All triplets have the same X, ***SLC1A3,*** and Y, ***MBP.*** Annotations for highlighted genes in this table are ***MBP*** (myelin basic protein), ***SLC1A3*** (solute carrier family 1 (glial high affinity glutamate transporter), member 3), ***MAG*** (myelin associated glycoprotein) ***IRF1*** (interferon regulatory factor 1), ***APOE*** (apolipoprotein E), ***PDGFA*** (platelet-derived growth factor alpha polypeptide), ***ST8SIA1*** (sialyltransferase 8A), ***SOX4*** (SRY (sex determining region Y)-box 4), ***SOX9*** (SRY (sex determining region Y)-box 9), ***HLA-DQB1*** (major histocompatibility complex, class II, DQ beta 1), ***EPHA2*** (EphA2). The p-value is estimated by permuting Z 1,000,000 times.

Supplementary Table 6: LA output X=SLC1A3, Y=MBP, Z=Any, (NCI_cDNA)

Z	LAP	PVAL	CC (L)	CC (H)	CC (H-L)	Z	LAP	PVAL	CC (L)	CC (H)	CC (H-L)
EML1	0.3344	1.0E-04	-0.36	0.47	0.83	MRPL22	-0.3119	2.0E-04	0.32	-0.47	-0.79
W92878	0.3152	2.3E-04	-0.44	0.77	1.21	CDK6	-0.3104	2.7E-04	0.45	-0.44	-0.89
PRKAB2	0.2992	4.0E-04	-0.54	0.47	1.01	**TRIM10**	-0.308	3.8E-04	0.49	-0.38	-0.86
PPP2R4	0.2987	4.1E-04	-0.51	0.37	0.88	MARS	-0.3074	2.7E-04	0.33	-0.65	-0.98
IGSF3	0.2983	5.0E-04	-0.44	0.35	0.79	LAPTM5	-0.305	4.8E-04	0.34	-0.40	-0.74
CKM	0.2911	5.9E-04	-0.32	0.28	0.60	LRRC61	-0.299	1.2E-03	0.42	-0.49	-0.91
CENPB	0.2903	7.2E-04	-0.32	0.32	0.65	**EVI2A**	-0.2972	5.3E-04	0.54	-0.50	-1.03
KIAA0748	0.289	6.6E-04	-0.38	0.32	0.70	**TAP2**	-0.2942	5.2E-04	0.63	-0.66	-1.29
HYAL2	0.2838	8.1E-04	-0.60	0.34	0.94	OSBP2	-0.2933	5.5E-04	0.34	-0.53	-0.87
AOC3	0.2812	8.7E-04	-0.45	0.52	0.97	USP4	-0.2931	5.7E-04	0.59	-0.75	-1.34
N99816	0.2804	9.6E-04	-0.44	0.41	0.85	ARHGEF7	-0.2931	5.3E-04	0.32	-0.36	-0.68
LOC126917	0.2778	9.9E-04	-0.81	0.35	1.16	PTK2B	-0.2927	5.6E-04	0.37	-0.63	-1.00
PFKL	0.2771	1.1E-03	-0.36	0.27	0.63	LOC285074	-0.2926	5.6E-04	0.36	-0.38	-0.74
GSTM3	0.2764	1.1E-03	-0.44	0.40	0.84	CDK6	-0.292	5.9E-04	0.31	-0.36	-0.67
COQ2	0.2753	1.1E-03	-0.67	0.34	1.01	RBM15B	-0.2917	5.2E-04	0.73	-0.50	-1.23
ICHTHYIN	0.2727	1.3E-03	-0.50	0.35	0.85	GART	-0.2897	6.3E-04	0.35	-0.46	-0.82
C14orf179	0.2726	1.2E-03	-0.56	0.50	1.07	YARS	-0.2875	6.7E-04	0.45	-0.64	-1.09
NA-4508	0.2682	1.7E-03	-0.51	0.40	0.91	RARRES2	-0.2846	8.2E-04	0.65	-0.45	-1.10
C14orf131	0.2666	1.6E-03	-0.48	0.26	0.74	FCN3	-0.2827	1.5E-03	0.26	-0.47	-0.73
DCXR	0.2614	3.1E-03	-0.35	0.36	0.71	FRMD4A	-0.2774	1.0E-03	0.30	-0.43	-0.73

Table S.6 In this table, only column Z is displayed. All triplets have the same X, ***MBP,*** and Y, ***SLC1A3.*** Annotations for highlighted genes in this table are ***MBP*** (myelin basic protein), ***SLC1A3*** (solute carrier family 1 (glial high affinity glutamate transporter), member 3), ***TRIM10*** (tripartite motif-containing 10; Located in HLA locus), ***EVI2A*** (ecotropic viral integration site 2A), ***TAP2*** (transporter 2, ATP-binding cassette, sub-family B (MDR/TAP); Candidate Gene;Located in HLA locus).

Table S.7: LA output X=SLC1A3 Y=HLA Family Z=Any (NCI_Affy)

Y	Z	LAP	PVAL	CC (L)	CC (H)	CC (H-L)	Y	Z	LAP	PVAL	CC (L)	CC (H)	CC (H-L)
HLA-DPA1	**PDGFRA**	0.5716	0	-0.65	0.69	1.34	HLA-DQA1	PTGS2	-0.539	0	0.60	-0.48	-1.08
HLA-C	H87358	0.5513	0	-0.84	0.76	1.60	HLA-DRB5	GABRA1	-0.537	0	0.68	-0.81	-1.49
HLA-C	PRB4	0.5498	0	-0.77	0.65	1.42	HLA-C	APOBEC3A	-0.537	0	0.64	-0.45	-1.09
HLA-DRB1	CD2	0.5452	0	-0.65	0.34	0.99	HLA-DQB1	IKZF4	-0.536	5.0E-06	0.73	-0.83	-1.56
HLA-DRB1	VIPR2	0.5417	0	-0.67	0.59	1.26	HLA-DQA1	IGFBP2	-0.529	0	0.38	-0.67	-1.05
HLA-DQA1	MARCKS	0.5381	0	-0.59	0.79	1.39	HLA-DRB5	NME3	-0.527	0	0.60	-0.86	-1.47
HLA-G	**PDGFRA**	0.5339	0	-0.65	0.58	1.23	HLA-A	STAT6	-0.521	1.6E-05	0.58	-0.65	-1.23
HLA-DRB1	TSC22D3	0.5338	1.0E-06	-0.61	0.61	1.22	HLA-DQA1	MAP4K2	-0.513	2.0E-06	0.37	-0.61	-0.99
HLA-A	**PDGFRA**	0.5297	1.1E-05	-0.57	0.42	0.98	HLA-DQB1	VCAN	-0.509	2.0E-06	0.52	-0.64	-1.17
HLA-F	**PDGFRA**	0.5289	0	-0.71	0.51	1.22	HLA-DRB5	R10882	-0.507	0	0.57	-0.67	-1.24
HLA-A	**PDGFRA**	0.5287	8.0E-06	-0.81	0.55	1.36	HLA-DQA1	NGFB	-0.505	3.0E-06	0.39	-0.61	-1.00
HLA-DRB1	IL13	0.5263	1.0E-06	-0.65	0.71	1.36	HLA-G	STAT6	-0.497	3.0E-06	0.60	-0.54	-1.14
HLA-G	**PDGFRA**	0.5223	0	-0.73	0.52	1.24	HLA-DRB5	H38679	-0.497	0	0.60	-0.45	-1.05
HLA-DQB1	MAP4K2	0.5221	2.0E-06	-0.71	0.69	1.41	HLA-DQA1	GPR3	-0.494	3.0E-06	0.46	-0.47	-0.93
HLA-DQA1	PLEK	0.5201	1.0E-06	-0.63	0.79	1.42	HLA-DRB5	H69878	-0.492	2.0E-06	0.71	-0.64	-1.34
HLA-C	KCNJ4	0.5187	0	-0.54	0.59	1.13	HLA-DRB5	VWA1	-0.491	1.0E-06	0.63	-0.67	-1.30
HLA-DRB1	TMEM149	0.5184	1.0E-06	-0.80	0.70	1.50	HLA-DQB1	PDGFRA	-0.49	8.0E-06	0.37	-0.69	-1.06
HLA-DQA1	**PDGFRA**	0.5147	0	-0.79	0.76	1.55	HLA-DOA	LIPA	-0.488	3.0E-06	0.54	-0.38	-0.92
HLA-A	FLT3LG	0.5033	2.7E-05	-0.82	0.66	1.48	HLA-G	STAT6	-0.488	9.0E-06	0.46	-0.67	-1.12
HLA-G	**PDGFRA**	0.5026	4.0E-06	-0.69	0.46	1.15	HLA-DQA1	PTPN2	-0.484	5.0E-06	0.48	-0.74	-1.22

Table S.7 In this table, only columns for Y and Z are displayed. All triplets have the same X, ***SLC1A3.*** Annotations for highlighted genes in this table are ***SLC1A3*** (solute carrier family 1 (glial high affinity glutamate transporter), member 3), ***HLA-F*** (major histocompatibility complex, class I, F), ***HLA-DQA1*** (major histocompatibility complex, class II, DQ alpha 1), ***HLA-G*** (HLA-G histocompatibility antigen, class I, G), ***HLA-E*** (major histocompatibility complex, class I, E), ***HLA-C*** (major histocompatibility complex, class I, C), ***HLA-DRB3*** (major histocompatibility complex, class II, DR beta 3), ***HLA-DOA*** (major histocompatibility complex, class II, DO alpha), ***HLA-DQB1*** (major histocompatibility complex, class II, DQ beta 1), ***HLA-DPB1*** (major histocompatibility complex, class II, DP beta 1), ***HLA-DRB5*** (major histocompatibility complex, class II, DR beta 5), ***PDGFRA*** (platelet-derived growth factor receptor, alpha polypeptide). The p-value is estimated by permuting Z 1,000,000 times.

Table S.8: LA output X=SLC1A3 Y=HLA Family Z=Any (NCI_cDNA)

Y	Z	LAP	PVAL	CC (L)	CC (H)	CC (H-L)	Y	Z	LAP	PVAL	CC (L)	CC (H)	CC (H-L)
HLA-DRB1	ADAR	0.491	6.0E-06	-0.62	0.42	1.04	HLA-E	**GMFB**	-0.5172	7.0E-06	0.57	-0.70	-1.26
HLA-DQA1	ERCC3	0.4836	4.0E-06	-0.61	0.44	1.05	HLA-DQA1	AGXT2L1	-0.514	2.0E-06	0.57	-0.76	-1.33
HLA-DRB1	GRK4	0.4776	0	-0.75	0.27	1.02	HLA-A	**GMFB**	-0.5108	1.0E-06	0.82	-0.38	-1.20
HLA-DRB1	TARBP1	0.4771	7.0E-06	-0.78	0.44	1.21	HLA-DQA1	IGSF3	-0.5011	1.0E-06	0.60	-0.61	-1.21
HLA-DQA1	RPL10	0.4756	1.8E-05	-0.51	0.54	1.04	HLA-DRA	MSRA	-0.4968	0	0.36	-0.64	-1.00
HLA-DRB1	HSPA14	0.4581	1.2E-05	-0.66	0.30	0.95	HLA-A	**GMFB**	-0.4862	0	0.76	-0.53	-1.29
HLA-DQA1	MARCH6	0.4576	3.9E-05	-0.80	0.71	1.52	HLA-DQA1	NLGN1	-0.4727	2.5E-05	0.75	-0.64	-1.39
HLA-DRB1	ZC3HAV1	0.4518	2.0E-05	-0.62	0.51	1.13	HLA-A	NA-4456	-0.4707	1.9E-05	0.58	-0.62	-1.19
HLA-DRB1	ME2	0.4517	1.6E-05	-0.71	0.56	1.28	HLA-E	NA-4456	-0.4706	4.5E-05	0.55	-0.66	-1.20
HLA-DMA	KIF21B	0.4454	1.0E-06	-0.74	0.57	1.31	HLA-B	**GMFB**	-0.4677	9.0E-06	0.86	-0.35	-1.21
HLA-DRB1	WNK2	0.4441	9.4E-05	-0.53	0.47	1.00	HLA-E	DDO	-0.4625	6.2E-05	0.61	-0.91	-1.52
BAT4	GRB14	0.4429	5.3E-05	-0.81	0.22	1.03	HLA-DQA1	FAM135B	-0.4593	4.5E-05	0.49	-0.47	-0.96
HLA-DRB1	ZYG11B	0.4425	2.5E-05	-0.62	0.59	1.21	HLA-DQA1	KIAA0999	-0.4573	2.0E-05	0.56	-0.80	-1.36
HLA-DQA1	GZMH	0.4404	1.7E-04	-0.53	0.61	1.14	HLA-A	**GMFB**	-0.457	2.3E-05	0.82	-0.78	-1.60
HLA-E	SLC2A5	0.4378	9.3E-05	-0.46	0.59	1.05	HLA-DQA1	LMOD1	-0.4555	4.0E-06	0.62	-0.71	-1.33
HLA-DQA1	KIF22	0.435	5.3E-05	-0.45	0.53	0.99	HLA-DQA1	AA027217	-0.4534	5.4E-05	0.57	-0.79	-1.36
HLA-DPA1	GRK4	0.4304	2.3E-05	-0.65	0.30	0.96	HLA-DQA1	R35024	-0.4516	6.8E-05	0.62	-0.85	-1.47
HLA-DMA	CG018	0.4302	3.0E-06	-0.87	0.21	1.08	HLA-DQA1	ARG1	-0.4516	3.2E-05	0.42	-0.81	-1.22
HLA-DPA1	PTDSS1	0.4271	1.5E-05	-0.79	0.50	1.30	BAT2	AA054500	-0.4487	7.0E-06	0.59	-0.45	-1.04
HLA-DRB1	PTPRA	0.426	9.5E-05	-0.81	0.80	1.62	HLA-DQB1	ACP5	-0.4431	0	0.47	-0.57	-1.04

Table S.8 In this table, only columns for Y and Z are displayed. All triplets have the same X, ***SLC1A3.*** Annotations for highlighted genes in this table are ***SLC1A3*** (solute carrier family 1 (glial high affinity glutamate transporter), member 3), ***HLA-F*** (major histocompatibility complex, class I, F), ***HLA-DQA1*** (major histocompatibility complex, class II, DQ alpha 1), ***HLA-G*** (HLA-G histocompatibility antigen, class I, G), ***HLA-E*** (major histocompatibility complex, class I, E), ***HLA-C*** (major histocompatibility complex, class I, C), ***HLA-DRB3*** (major histocompatibility complex, class II, DR beta 3), ***HLA-DOA*** (major histocompatibility complex, class II, DO alpha), ***HLA-DQB1*** (major histocompatibility complex, class II, DQ beta 1), ***HLA-DPB1*** (major histocompatibility complex, class II, DP beta 1), ***HLA-DRB5*** (major histocompatibility complex, class II, DR beta 5), ***HLA-DMA*** (major histocompatibility complex, class II, DM alpha), ***BAT2*** (HLA-B associated transcript 2), ***BAT4*** (HLA-B associated transcript 4), ***GMFB*** (glia maturation factor, beta). The p-value is estimated by permuting Z 1,000,000 times.

Table S.9: Highest correlated genes of SLC1A3 (GNF_2004)

X	Y	CORR
SLC1A3	Hs.7309	0.727857
SLC1A3	**NTRK2**	0.722238
SLC1A3	TU3A	0.707924
SLC1A3	TU3A	0.701041
SLC1A3	**CTNND2**	0.697423
SLC1A3	DNER	0.69232
SLC1A3	TM4SF2	0.689496
SLC1A3	NEBL	0.687918
SLC1A3	LMO3	0.680221
SLC1A3	ATP1A2	0.673797
SLC1A3	SPARCL1	0.671678
SLC1A3	**PKP4**	0.670142
SLC1A3	KIAA0644	0.669571
SLC1A3	STMN2	0.668745
SLC1A3	DTNA	0.668604
SLC1A3	KIAA1102	0.668579
SLC1A3	ANK2	0.658617
SLC1A3	CAP2	0.6585
SLC1A3	AA218868	0.65786
SLC1A3	DAAM2	0.65734

Table S.9 In this table, the genes highly correlated with ***SLC1A3*** are displayed. Annotations for genes highlighted in this table are ***SLC1A3*** (solute carrier family 1 (glial high affinity glutamate transporter), member 3), ***NTRK2*** (Neurotrophic tyrosine kinase, receptor type 2; PMID: 11834594), ***PKP4*** (plakophilin 4, Members of the p120(ctn)/plakophilin subfamily of Armadillo-like proteins, including CTNND1, CTNND2, PKP1, PKP2, PKP4, and ARVCF. PKP4 may be a component of desmosomal plaque and other adhesion plaques and is thought to be involved in regulating junctiona), ***CTNND2*** (catenin (cadherin-associated protein), delta 2 (neural plakophilin-related arm-repeat protein)).

Table S.10: Highest correlated genes of MBP (GNF_2004)

Y	Z	CORR
MBP	**PLP1**	0.766726
MBP	HSPA12A	0.747412
MBP	**KLK6**	0.742093
MBP	KIAA0644	0.726952
MBP	**PMP2**	0.72539
MBP	**ANK2**	0.724356
MBP	**SYT1**	0.722174
MBP	PLEKHB1	0.721199
MBP	ATP1A2	0.716834
MBP	DNM3	0.713579
MBP	APLP1	0.712538
MBP	INA	0.711727
MBP	MBP	0.710892
MBP	MBP	0.710892
MBP	**CTNND2**	0.710164
MBP	DNCI1	0.709571
MBP	DPP6	0.708166
MBP	TUBB5	0.708151
MBP	CAMK2B	0.707095
MBP	TM4SF11	0.707006

Table S.10 In this table, the genes highly correlated with ***MBP*** are displayed. Annotations for genes highlighted in this table are ***MBP*** (myelin basic protein), ***PLP1*** (proteolipid protein 1 (Pelizaeus-Merzbacher disease, spastic paraplegia 2, uncomplicated)), ***KLK6*** (kallikrein 6 (neurosin, zyme)), ***PMP2*** (peripheral myelin protein 2), ***ANK2*** (ankyrin 2, neuronal), ***SYT1*** (synaptotagmin 1), ***CTNND2*** (catenin (cadherin-associated protein), delta 2 (neural plakophilin-related arm-repeat protein)).
