# Supplementary material for: Finding disease candidate genes by liquid association
Source: Genome Biol. 2007 Oct 4;8(10):R205. doi: 10.1186/gb-2007-8-10-r205 (PMC2246280; doi:10.1186/gb-2007-8-10-r205)
Supplement: Additional data file 2 — Supplementary text detailing the additional data analyses mentioned in the text is provided. [file gb-2007-8-10-r205-S2.doc]

# Supplementary Text

## Text 1. Alzheimer Disease genes: an illustrative use of LA.

Liquid association(LA) offers a scoring system to guide a genome-wide search for critical cellular players that may interfere with the coexpression of a pair of genes, thereby weakening their overall correlation. A basic use of LA is to start with two functionally related genes X, Y. Consider the Alzheimer disease hall mark gene, APP(amyloid beta precursor protein). The amyloid beta peptide found in the plagues of Alzheimer brains is generated from the consecutive proteolytic cleavage of APP by the beta secretase at the N terminus and by the gamma secretase at the C terminus. The normal physiological role of APP was first linked to the control of gene expression in (Cao and Sudhof 2001), where the carboxyl-terminal intracellular fragment of APP was found to interact with the nuclear adaptor protein Fe65 (encoded by APBB1) and the histone acetyltransferase Tip60 (encoded by HTATIP). Using the NCI_cDNA database, the profiles of APBB1 and HTATIP are compared with that of APP. The correlations (-.06, -.27 respectively) are quite low. In search of genes which may play a role in weakening the correlation, set X= APP, Y=APBB1 to compute the LA score LA(X,Y|Z) for each of the 9076 transcripts in the database. After ranking all 9076 LA scores, a beta-site APP-cleaving enzyme BACE2 is found to be in the short list of 20 genes with best LA scores. We next apply LA again to the pair X=APP, Y=HTATIP. This time PSEN1 (presenilin 1), which is a major component of gamma-secretase, is found to have the second highest LA score! For more discussion, see Li et al. (2004). Our website provide on-line calculation for any pair of genes; <http://kiefer.stat.ucla.edu/lap>.

Cao X, Sudhof TC. A transcriptionally [correction of transcriptively] active complex of APP with Fe65 and histone acetyltransferase Tip60. *Science*. **293**:115-20 (2001). Erratum in: *Science* **293**:1436 (2001).

Li, K.C., Liu, C.T, Sun, W.,Yuan, S, & Yu,T. A system for enhacing genome-wide coexpression dynamics study. *PNAS* **101**, 15561-15566 (2004).

## Text2 – Distribution of LA scores using MBP as the scouting gene.

Using MBP as the scouting gene Z, we compute the LA score L(X,Y|Z) for every pair of genes in the NCI_cDNA dataset. This generates around 49 millions of LA scores. The distribution is shown here. The extreme values are shown in the zoom-in plots.

##
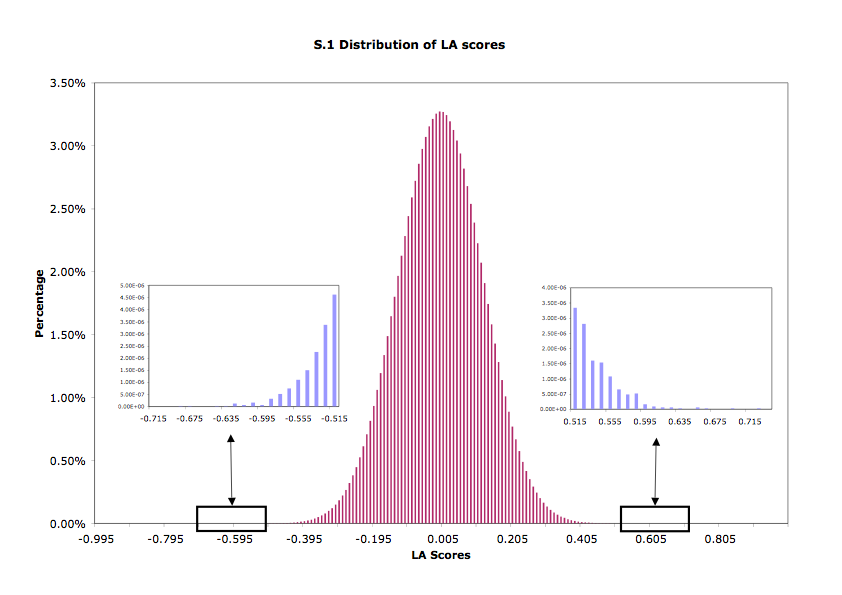


## Text 3 – Significance of the MBP’s scouting result

Because the expression profile of MBP in the NCI_cDNA data set has two missing values, only 60-2 (=58) conditions are used in computing the LA score. We consider the MBP profile itself, after normal score transformation, to be representative of the state variable correlated with MBP. To access the significance of the scouting result for MBP, we would like to generate a reference set of pseudo state profiles and evaluate their LA scouting performances. This is carried out in the following way.

1. **Generation of pseudo cellular state variable profiles.** We generate the pseudo state variable profiles, by permuting the MBP state variable. Repeat this procedure for 1000 times.
2. **Find the LA scouting result.** For each generated pseudo state profile, we perform the LA scouting procedure that computes the LA statistics for all 49 million pairs, using the generated state profile as the scouting gene profile. For sorting by LA scores, the top (most positive) 25 LA scores and the bottom (most negative) 25 LA scores are saved.
3. **Compare the LA score for MBP with the reference LA score distribution.** For the 1000 randomly generated pseudo state profiles, we found that there are only 16 of them have a higher 25th top LA score than that observed in MBP’s scouting result.

The above study indicates a significance level of about 1.6% for the LA scouting report of MBP. An important assumption we have made is that the randomly generated state profiles overall are less likely to represent a biologically meaningful state. However, it is still possible that some computer-generated states may happen to resemble biologically meaningful states and it is better to exclude such states as much as possible when creating the reference set of pseudo state profiles. To proceed in this direction, the following additional step is taken:

### Compute an index of closeness to gene expression profiles. For each computer-generated state profile, we compute the correlation between the state profile and the expression profile of each gene in NCI_cDNA data. We use the 5^th^ highest correlation as the index of how close to gene expression profiles the simulated state profile is.

We reason that if a simulated state profile is biologically meaningless, then it is less likely to find genes correlated to it. Consequently, poorer LA scores can be expected for the simulated state profiles that have lower values of closeness measure computed at step 4. This is confirmed by inspecting the scatterplot of the closeness index and the 25^th^ LA score, plotted in Supplementary Figure S.2. In this figure, we see a clear positive trend for the top LA score and a negative trend for the bottom LA score.

For the 16 pseudo state profiles that have better LA score than MBP, they do show larger values in the closeness index. In fact, if we use only the 500 pseudo states that show the lower values of the closeness index state to create the reference distribution of LA scores, then only 2 cases have better LA scores, leading to the improvement of significance to 2/500 = 0.4%.


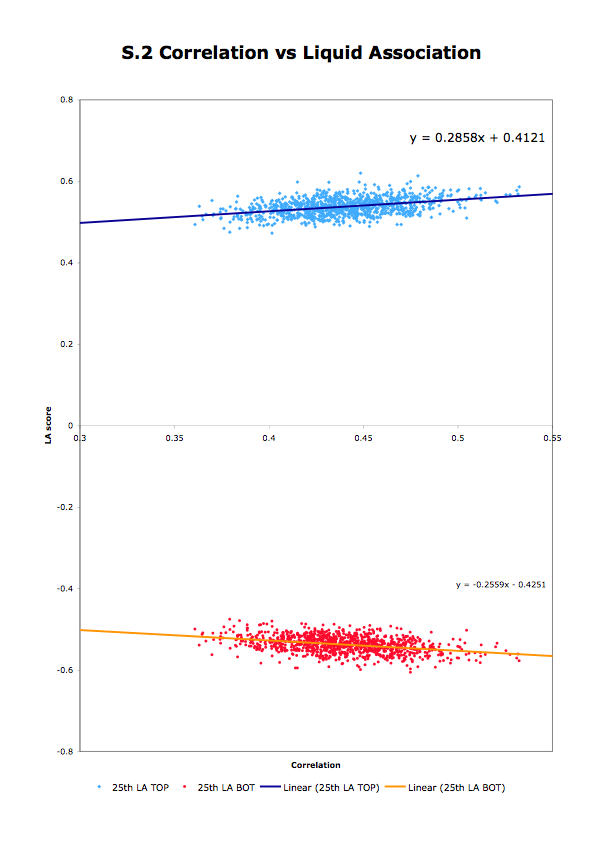


Figure S.2. The horizontal axis shows the closeness index of a computer-generated state profile. The vertical axis shows the 25^th^ top LA score (upper panel) and the bottom 25^th^ LA score (lower panel) for the corresponding state profile, A positive (negative) trend is observed in the upper (lower) panel.

***Text 4 – Correlation Matrix for genes paired with MBP and A2M.***

***Correlations between the genes that are paired with A2M in the LAP output of MBP are computed. All of them are found to be positively correlated. The correlation values higher than 0.30, 0.39 and 0.463 are statistically significant at level 0.01, 0.001 and 0.0001 respectively, based on a permutation test of 1,000,000 runs.***

|  | ZBTB40 | ***MPDZ*** | AA040811 | ***TRIB2*** | C2orf32 | CPEB2 | N66535 |
| --- | --- | --- | --- | --- | --- | --- | --- |
| ZBTB40 | 1.00 | 0.43 | 0.44 | 0.89 | 0.67 | 0.52 | 0.73 |
| ***MPDZ*** | 0.43 | 1.00 | 0.10 | 0.30 | 0.37 | 0.29 | 0.39 |
| AA040811 | 0.44 | 0.10 | 1.00 | 0.48 | 0.54 | 0.54 | 0.46 |
| ***TRIB2*** | 0.89 | 0.30 | 0.48 | 1.00 | 0.71 | 0.54 | 0.77 |
| C2orf32 | 0.67 | 0.37 | 0.54 | 0.71 | 1.00 | 0.52 | 0.96 |
| CPEB2 | 0.52 | 0.29 | 0.54 | 0.54 | 0.52 | 1.00 | 0.52 |
| NA-4095 | 0.73 | 0.39 | 0.46 | 0.77 | 0.96 | 0.52 | 1.00 |
| ZNF598 | 0.42 | 0.36 | 0.53 | 0.55 | 0.45 | 0.39 | 0.46 |
| C11orf41 | 0.50 | 0.41 | 0.37 | 0.39 | 0.35 | 0.33 | 0.32 |
| ***LYST*** | 0.56 | 0.29 | 0.59 | 0.60 | 0.63 | 0.66 | 0.61 |
| KIAA1539 | 0.42 | 0.37 | 0.57 | 0.44 | 0.49 | 0.49 | 0.45 |
| ***CHM*** | 0.41 | 0.08 | 0.38 | 0.44 | 0.46 | 0.36 | 0.49 |
| C1orf61 | 0.46 | 0.46 | 0.36 | 0.42 | 0.54 | 0.42 | 0.53 |
| LUZP1 | 0.36 | 0.36 | 0.41 | 0.34 | 0.34 | 0.48 | 0.28 |
| TMF1 | 0.47 | 0.15 | 0.48 | 0.52 | 0.53 | 0.47 | 0.52 |

|  | ZNF598 | C11orf41 | ***LYST*** | KIAA1539 | ***CHM*** | C1orf61 | LUZP1 | TMF1 |
| --- | --- | --- | --- | --- | --- | --- | --- | --- |
| ZBTB40 | 0.42 | 0.50 | 0.56 | 0.42 | 0.41 | 0.46 | 0.36 | 0.47 |
| ***MPDZ*** | 0.36 | 0.41 | 0.29 | 0.37 | 0.08 | 0.46 | 0.36 | 0.15 |
| AA040811 | 0.53 | 0.37 | 0.59 | 0.57 | 0.38 | 0.36 | 0.41 | 0.48 |
| ***TRIB2*** | 0.55 | 0.39 | 0.60 | 0.44 | 0.44 | 0.42 | 0.34 | 0.52 |
| C2orf32 | 0.45 | 0.35 | 0.63 | 0.49 | 0.46 | 0.54 | 0.34 | 0.53 |
| CPEB2 | 0.39 | 0.33 | 0.66 | 0.49 | 0.36 | 0.42 | 0.48 | 0.47 |
| NA-4095 | 0.46 | 0.32 | 0.61 | 0.45 | 0.49 | 0.53 | 0.28 | 0.52 |
| ZNF598 | 1.00 | 0.41 | 0.50 | 0.54 | 0.22 | 0.21 | 0.22 | 0.29 |
| C11orf41 | 0.41 | 1.00 | 0.30 | 0.40 | 0.27 | 0.31 | 0.51 | 0.44 |
| ***LYST*** | 0.50 | 0.30 | 1.00 | 0.55 | 0.34 | 0.47 | 0.39 | 0.44 |
| KIAA1539 | 0.54 | 0.40 | 0.55 | 1.00 | 0.38 | 0.23 | 0.56 | 0.34 |
| ***CHM*** | 0.22 | 0.27 | 0.34 | 0.38 | 1.00 | 0.46 | 0.34 | 0.43 |
| C1orf61 | 0.21 | 0.31 | 0.47 | 0.23 | 0.46 | 1.00 | 0.19 | 0.35 |
| LUZP1 | 0.22 | 0.51 | 0.39 | 0.56 | 0.34 | 0.19 | 1.00 | 0.39 |
| TMF1 | 0.29 | 0.44 | 0.44 | 0.34 | 0.43 | 0.35 | 0.39 | 1.00 |

***Text 5 – Computing the effect size (correlation change for each triplet)***

It would be more transparent to assess the effect of liquid association by inspecting the LA activity plot ; see Figure 4 in the main text for an example. We now explain how the two slopes are computed. The aim here is to find the cutoff points for three group of conditions, the high expression group, the median expression group, and the low expression group. Once the cutoff points are decided, we simple use the linear regression to find the trend. Two algorithms are developed for this purpose. Let Z be the mediator gene and X,Y be the pair of genes that Z mediates. Normal score transformations are taken first before applying these algorithms

(A). **MLE algorithm**. This method treats this problem as a mixture normal model. For each pair of cutoff values, C1, C2, we compute the goodness of fit for the linear models Y= a+ bX+ error, for three groups, Z<C1, C1<Z<C2, Z>C2, separately by the standard sum of squares. The combined goodness of fit will be the sum from three sum of squares. The cutoff values that give o the smallest total sum of squares are the final output.

(B). **DISCRETE algorithm**. For a given pair of cutoff values,C1, C2, we compute the difference between the total of X*Y for the first group Z<C1 and that for the third group Z>C2. The pair of C1 and C2 that leads to the maximum absolute value of the difference is the final output.

For most cases, these two methods lead to similar results. In our report for each LA output table, we give the one that shows the better correlation change.
